# Recent Progress in Silicon-Based Slow-Light Electro-Optic Modulators

**DOI:** 10.3390/mi13030400

**Published:** 2022-02-28

**Authors:** Changhao Han, Ming Jin, Yuansheng Tao, Bitao Shen, Xingjun Wang

**Affiliations:** 1 State Key Laboratory of Advanced Optical Communications System and Networks, School of Electronics, Peking University, Beijing 100871, China; chhan@pku.edu.cn (C.H.); mjin@pku.edu.cn (M.J.); ystao@pku.edu.cn (Y.T.); btshen@pku.edu.cn (B.S.); 2 Frontiers Science Center for Nano-Optoelectronics, Peking University, Beijing 100871, China; 3 Peng Cheng Laboratory, Shenzhen 518055, China; 4 Peking University Yangtze Delta Institute of Optoelectronics, Nantong 226010, China

**Keywords:** silicon photonics, slow-light effect, electro-optic modulators, compact footprint

## Abstract

As an important optoelectronic integration platform, silicon photonics has achieved significant progress in recent years, demonstrating the advantages on low power consumption, low cost, and complementary metal–oxide–semiconductor (CMOS) compatibility. Among the different silicon photonics devices, the silicon electro-optic modulator is a key active component to implement the conversion of electric signal to optical signal. However, conventional silicon Mach–Zehnder modulators and silicon micro-ring modulators both have their own limitations, which will limit their use in future systems. For example, the conventional silicon Mach–Zehnder modulators are hindered by large footprint, while the silicon micro-ring modulators have narrow optical bandwidth and high temperature sensitivity. Therefore, developing a new structure for silicon modulators to improve the performance is a crucial research direction in silicon photonics. Meanwhile, slow-light effect is an important physical phenomenon that can reduce the group velocity of light. Applying slow-light effect on silicon modulators through photonics crystal and waveguide grating structures is an attractive research point, especially in the aspect of reducing the device footprint. In this paper, we review the recent progress of silicon-based slow-light electro-optic modulators towards future communication requirements. Beginning from the principle of slow-light effect, we summarize the research of silicon photonic crystal modulators and silicon waveguide grating modulators in detail. Simultaneously, the experimental results of representative silicon slow-light modulators are compared and analyzed. Finally, we discuss the existing challenges and development directions of silicon-based slow-light electro-optic modulators for the practical applications.

## 1. Introduction

With the development of the modern society, the communication technologies with massive information transmission requirements have risen rapidly, such as big data, artificial intelligence, cloud computing, and 5G. The capacity demand of the network continues to increase, which means higher requirements for the transmission and process speed in communication systems. Optical communication has the advantages of high speed, large capacity, low loss, anti-electromagnetic interference, and low crosstalk, while the rapid development in optical communication relies on the breakthrough in basic photonic devices [1]. However, the optical module components in traditional optical communication systems are composed of discrete components, and the complexity, cost, and power consumption increase rapidly with the continuous expansion of network capacity. Integrating various devices on a single chip is the current trend of optical communication, which is similar to microelectronics. The photonics integration can achieve high density, low cost, and low energy consumption, which is significant to satisfy the communication requirements of the future information society [2].

As a significant integrated optoelectronic technology, silicon photonics demonstrates the advantages of low power consumption, low cost, and CMOS compatibility, and is receiving more and more attention from both academia and industry [3]. Nowadays, silicon photonics has been applied in optical communication networks and data centers, and a lot of progress has been made in recent years under the continuous innovation of researchers [4,5,6,7,8]. As a key active device to complete the conversion of electrical signals to optical signals, silicon modulators exhibit a high modulation rate and low-cost potential, which makes it a prospect for a broad range of applications. At present, the most commonly used modulation principle in silicon modulators is the plasma dispersion effect, which means the real and imaginary parts of the refractive index will change by controlling the carrier concentration in silicon [9]. According to their structure, silicon modulators can be divided into silicon Mach–Zehnder modulators (MZMs) and silicon micro-ring modulators (MRMs). The advantages of conventional silicon MZMs include good thermal stability, small chirp, large extinction ratio, wide operating wavelength range, and low sensitivity to fabrication error and operating temperature, which demonstrate an applicability for commercial use. However, the conventional silicon MZM is hindered by large footprint, high driving voltage, and high power consumption. Although the footprint of conventional silicon MZMs is much smaller than that of traditional lithium niobate modulators, most of them are still in the millimeter order, and there is still a distance from the requirements of large-scale optoelectronic integration [10,11,12]. Meanwhile, the silicon MRM has the advantages of small footprint and lower power consumption, but with chirp, narrow optical bandwidth, and high requirements of manufacture process and operation temperature. Due to the high temperature sensitivity of silicon MRMs, a temperature control module is often required in practical work, which requires a lot of extra energy consumption [13,14,15]. In fact, it can be seen that both silicon MZMs and MRMs have their own advantages and disadvantages, and it is difficult to satisfy all requirements of practical work simultaneously. Despite their performance limitations, these all-silicon devices can be fabricated under the CMOS process, which provides a prerequisite for large-scale practical applications. In addition, more and more silicon-based hybrid modulators have been developed recently, such as silicon-based lithium niobate (LN) modulators, silicon-based polymer modulators, and silicon-based graphene modulators [16,17,18]. These modulators rely on electro-optical (EO) effect of new materials and generally exhibit excellent performance, such as high bandwidth, high modulation efficiency, and extremely small footprint, but the manufacturing process is complex and is not compatible with the CMOS fabrication process. Therefore, developing a new structure for silicon modulators to improve the performance and overcome the limitations under CMOS process is a significant research direction in silicon photonics. 

Slow-light effect is an important physical phenomenon that can reduce the group velocity of light by increasing the group refractive index [19]. The slow-light effect can be obtained by a series of optical structures, such as photonic crystal and waveguide grating, thereby increasing the interaction between light and the modulation region, improving the modulation efficiency of the silicon modulator to obtain a more compact footprint [20]. It is particularly worth noting that this type of structure can be fabricated under the CMOS process, which is much more reliable and simpler than that of various silicon-based hybrid modulators, providing the foundation for large-scale and widespread applications.

In this paper, we focus on silicon-based slow-light electro-optic modulators. Beginning from the basic principle of slow-light effect, we review the progress of silicon photonic crystal modulators and silicon waveguide grating modulators in detail. Meanwhile, the representative experimental results of silicon slow-light modulators under CMOS process are compared and analyzed. Finally, the current challenges and the future development direction of silicon-based slow-light electro-optic modulators are summarized.

## 2. Principle of Slow-Light Effect

### 2.1. Basic Theory of Slow-Light Effect

The basic physics theory of the slow-light effect is to reduce the group velocity of light by increasing the group refractive index. Considering a light propagating with the wave number k and angular frequency ω, its group velocity $v_{g}$ is inversely proportional to the first-order dispersion ${\left(dk/d\omega \right)}^{-1}$ by

(1) $$v_{g}=\frac{1}{dk/d\omega }=\frac{c}{n_{g}}\;$$

where *c* is the velocity of light in vacuum and $n_{g}$ is the group index defined as $c/v_{g}$.

**Figure 1:**
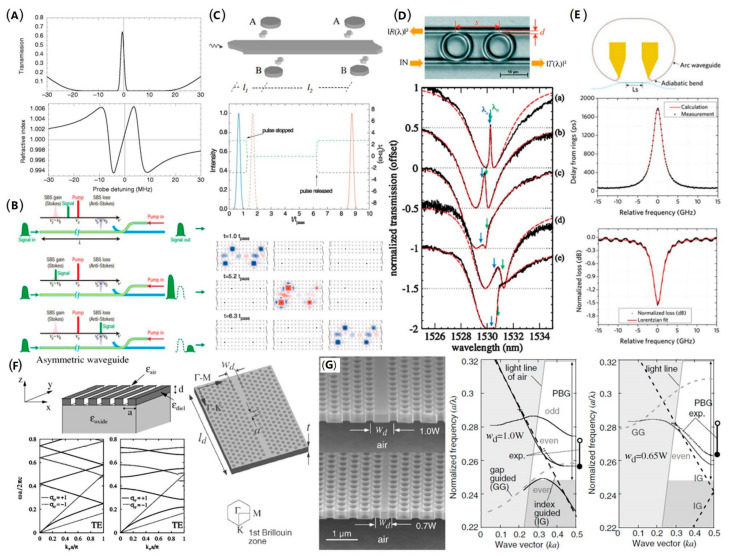
Different strategies for slow light. (**A**) Slow light down to 17 m/s by employing the electromagnetically induced transparency (EIT)in an ultracold gas of sodium atoms [22]. (**B**) Principle of slow light based on the stimulated Brillouin scattering (SBS) [21]. When a pump at frequency $\nu_{p}$ is injected into a waveguide, a gain peak and a loss peak will be produced at frequency $\nu_{p}-\nu_{b}$ (stokes frequency) and $\nu_{p}+\nu_{b}$ (anti-stokes frequency), respectively. Due to the gain (loss) peak, the signal at stokes (anti-stokes) frequency will undergo the slow (fast) light effect. The stokes frequency shift is directly relative to acoustic modes supported by the structure. (**C**) The all-optical analog of EIT by placing micro-disks around a strip waveguide [26]. (**D**) The on-chip all-optical analogue to EIT by cascading two ring resonates [27]. (**E**) A time delay realized by an all-pass ring resonant [28]. When meeting the resonance condition, the light will be stored in the ring. (**F**) A one-dimensional photonic crystal slab and its band diagrams [31]. By tuning the duty cycle of the grating, a strong coupling between the forward propagated mode and the backward propagated mode will be generated, resulting in an anti-crossing at the boundary of the brillouin zone. The anti-crossing will change the dispersion, and a strong slow-light effect can be induced. (**G**) A slow-light waveguide consisting of a line-defect in the two-dimensional photonic crystal slab [32]. The band diagrams show the anti-crossing between the index-guided mode and the gap-guided mode, where a slow-light effect is generated.

For $k=\varpi /\left(c/n\right)$, the group index can be expressed as

(2) $$n_{g}=c\frac{dk}{d\omega }=c\frac{d\left(n\omega \right)}{d\omega }=n+\omega \frac{dn}{d\omega }$$

where *n* is the material index. Regardless of the materials and optical structures, the material index itself will not change significantly. The major contribution of the slow-light effect is related with the first-order dispersion dn/dω. Inspired by the Kramers–Kronig transformation, a large first-order dispersion is always correlated with the narrow spectral gain or loss, and a large group index change can be realized by [21]

(3) $$∆n_{g}=\frac{\gamma c}{2\pi ∆\nu }$$

where $∆n_{g}$ indicates the change of group index and ∆ν is the linewidth of the gain or loss resonance. γ is the gain/loss coefficient. Various strategies can be employed to induce a narrow spectral gain or loss. One method is based on the electromagnetically induced transparency (EIT) in a vapor or a solid at, in most cases, cryogenic temperatures (Figure 1A) [22]. In addition, the optical nonlinear effect [23,24,25] can induce gain or loss at a certain frequency range. For example, the stimulated Brillouin scattering (SBS) [21], where remarkable photon and phonon interaction take places when a strong pump light is injecting into a nonlinear media, can generate narrow-spectrum gain around the stokes frequency and loss around the anti-stokes frequency, as shown in Figure 1B. 

Despite optical gain or loss generated by material properties, a strong slow-light effect can be realized by designed optical structures. The slow light based on EIT can also be realized by delicate placing of multiple micro-disks around a single waveguide (Figure 1C) [26], or by tuning two coupled resonators (Figure 1D) [27]. Ring resonate is a sort of common optical structure for slowing light, especially for integrated optical circuits. Figure 1E shows a typical all-pass ring resonate [28]. Meeting the resonance condition [29], a standing wave can be generated in the ring, regraded as a sort of stopped light.

Considering an optical waveguide, the propagation of light will be quite different from that in a bulk material. Among all the waveguides, the photonic crystal waveguides, consisting of period arrayed structures, are the most commonly used slow-light waveguide. Analogue to the electronic band theory in crystals, the photonic band theory [30] is the most powerful tool for the analysis of photonic crystals. Figure 1F shows a one-dimensional period structure and its band diagram [31]. As a coupling between forward and backward propagated waves caused by the period structure, a photonic band gap is generated due to the anti-crossing. The anti-crossing will intensively influence the dispersion around the crossing point, and a strong slow-light effect is produced. Similar effects can be found for two-dimensional photonic crystal waveguides where anti-crossing between index-guided mode and gap-guided mode (Figure 1G) [32] or even mode and odd mode [33], which can be employed to tuning the band diagram, resulting in zero-dispersion slow light or large band of slow light.

In general, the slow-light effect is related with the existence of the narrow spectral gain or loss, which can be induced by the material properties or the optical structures. Except for the above, medias with negative electromagnetic parameters can produce giant slow-light effect in a broad band [34]. However, the high loss blocks its application for modulator, which is also an important issue that needs to be solved. 

### 2.2. Basic Structure of Slow-Light Modulators

To apply the slow-light effect on silicon modulators, it is necessary to investigate the slow-light waveguides, which comprise the basis structure of the device. In fact, the waveguide is the fundamental unit in the modulator, and the slow-light waveguides are the key component to generate slow-light effect. The slow-light waveguides can be divided into photonic crystal waveguides and waveguide grating slow-light waveguides according to the different structures. Therefore, studying and optimizing the properties of slow-light waveguides is an important stage in the design and fabrication of complete silicon slow-light modulators.

**Figure 2:**
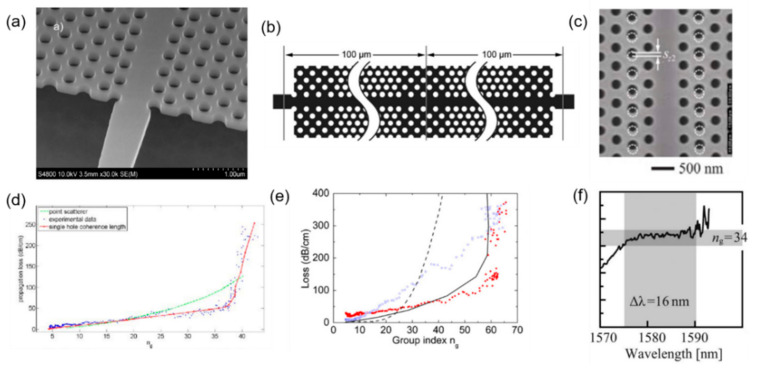
The structure and properties of photonic crystal waveguides. (**a**) SEM of a photonic crystal waveguide [35]. (**b**) The scheme of the slow–fast–slow mode conversion interface in the photonic crystal waveguide [36]. (**c**) SEM of fabricated photonic crystal waveguide with longitudinal lattice shifts in second rows [37]. (**d**) Loss as a function of group index in the manufactured photonic crystal waveguide [35]. (**e**) Loss of the photonic crystal waveguide as a function of group index with (red) and without (blue) slow–fast–slow mode conversion interface [36]. (**f**) Group index spectra for the photonic crystal waveguide with second-row longitudinal shifts [37].

Slow-light waveguides have larger loss than do conventional waveguides, and as the group index of refraction increases, the loss will also increase. For the slow-light effect to be applied in a practical condition, the large loss is a limiting factor that needs to be optimized. In 2010, L. O’Faolain et al. proposed a theoretical model for the loss of photonic crystal waveguides. The SEM micrograph of the photonic crystal waveguide is shown in Figure 2a. In this model, the entire hole will contribute to the scattering coherently. Based on the theory, they designed and fabricated low-loss photonic crystal waveguides (Figure 2d). The model can further reduce the loss of slow optical waveguides under existing process conditions. Meanwhile, they illustrated the importance of the loss per unit time metric for measuring the quality of slow-light waveguides. [35]. In 2012, J. Li et al. proposed a method by introducing a mode conversion interface of “slow–fast–slow” to reduce the scattering loss in lithographic stitching errors for photonic crystal waveguides with slow-light effect, as shown in Figure 2b. Based on this mode conversion, they reduced the waveguide loss from 320 dB/cm to 130 dB/cm at the group index of 60 (Figure 2e). Simultaneously, the length of the waveguide they fabricated by electron beam lithography reached 800 mm, which is much longer than the limited writing field of the electron beam lithography tool, thus the manufacturing difficulty of high-performance photonic crystal waveguides can be reduced [36].

In the practical operation, holding a large optical bandwidth is very important for slow-light waveguides and a wider working wavelength corresponds to a large working range. On the premise of controlling loss, the group refractive index and bandwidth need to be adjusted to obtain slow-light waveguides of higher performance for further application. In 2015, T. Tamura et al. calculated the photonic bands of the waveguide modes to study the influence of lattice-shifted structures on slow-light effect (Figure 2c). The photonic crystal waveguides are cladded by silica, which is fabricated under CMOS process. For each lattice shift, they found the second rows shifts, and the mixed shifts of the first and third rows bring in the slow-light effect with group index higher than 30. Meanwhile, this structure will hold a large bandwidth over 10 nm, which is suitable for practical application. Specifically, they observed the group index of 34 and the bandwidth of 16 nm (Figure 2f). However, this also illustrates that the lattice shift in the photonic crystal structure has a significant impact on the waveguide performance, indicating that photonic crystal waveguides still have relatively high requirements during the manufacturing process [37].

**Figure 3:**
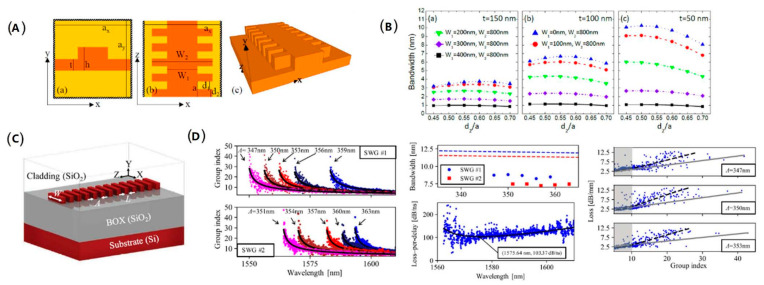
The structure and properties of silicon grating waveguides. (**A**) Cross-section for different views of the grating waveguide [38]. (**B**) Slow-light bandwidth with different parameters [38]. (**C**) Scheme of the subwavelength grating waveguide on SOI platform [39]. (**D**) Group index, slow-light bandwidth, loss-per-delay figure of merit, and loss of the subwavelength grating waveguides [39].

The silicon grating waveguides, as another periodic waveguide structure with slow-light effect, possess a simpler configuration than that of photonic crystal waveguides. In 2018, M. Passoni et al. developed a systematic analysis to investigate the photonic bands and group index in silicon grating waveguides. The scheme of the grating waveguide structure is illustrated in Figure 3A. They combined numerical methods and perturbation theory to demonstrate the slow-light performances with the change of geometric parameters. When the internal waveguide width and cladding silicon thickness are reduced, the slow-light bandwidth increases obviously. For instance, when the internal waveguide width gets to zero, the slow-light bandwidth reaches maximum. When the cladding silicon thickness reduces from 150 to 50 nm, the bandwidth increases from 3 nm to 10 nm (Figure 3B) [38]. In 2020, P. Jean et al. studied the slow-light effect in subwavelength grating waveguides experimentally on the silicon-on-insulator (SOI) platform, as shown in Figure 3C. By analytical modeling and 3D FDTD, they conducted detailed numerical study. The grating waveguides were fabricated by electron beam lithography process, and the resulting metrics included the large group index of 47.74, large slow-light bandwidth of 8.82 nm, loss-per-delay figure of merit of 103.37 dB/ns, and low loss of 12.5 dB/mm for the maximum group index (Figure 3D). Meanwhile, they demonstrated that controlling the design parameters will change the operation region of slow light over a large wavelength range. Based on the SOI platform, the subwavelength grating waveguides with slow-light effect have the advantage of compactness and are easier to manufacture, which makes them suitable for application in integrated optoelectronic devices [39].

Taking the advantages of slow-light effect, the slow-light modulator can be constructed on the basis structure of the slow-light waveguides. The group velocity of the light in slow-light waveguides is reduced, and the light–matter interaction in the modulation region can be enhanced, thereby enhancing the modulation capability of the modulator [20]. Modulators composed of slow-light waveguides are mostly based on the principle of phase modulation and the structure of the Mach–Zehnder (MZ) interferometer, which will be discussed in detail in the following sections. Slow-light waveguides are introduced to achieve higher modulation efficiency than that of conventional silicon MZMs. Therefore, the modulation effect that conventional modulators need long modulation region to achieve can be accomplished in a short length for slow-light modulators. Simultaneously, due to the large optical bandwidth of slow-light waveguides, the slow-light modulators can achieve a larger wavelength operating range than that of silicon MRMs, making them more suitable for practical work. In terms of materials, as discussed above, the ability to be fabricated under CMOS process is the core advantage of silicon photonic crystal waveguides and silicon grating waveguides. Most silicon-based slow-light electro-optic (EO) modulators are all-silicon modulators, which have the core advantage of CMOS compatibility, and overcome the shortcomings of conventional silicon MZMs and MRMs. Meanwhile, the slow-light waveguide structure can also be fabricated using other materials such as lithium niobite (LN), polymers, and graphene. These excellent EO materials can combine with silicon substrate and constitute silicon-based hybrid slow-light modulators to achieve higher breakthrough in performance.

## 3. Silicon Photonic Crystal Modulators

Photonic crystal is also called photonic band gap material. From the perspective of material structure, photonic crystal is a kind of artificially designed and fabricated crystal with periodic dielectric structures. When an electromagnetic wave propagates in a photonic band gap material, it is modulated due to Bragg scattering, and the energy of the electromagnetic wave forms an energy band structure. A band gap appears between the energy bands, which is the photonic band gap. Photons with energies within the photonic band gap cannot enter the crystal [40]. Two-dimensional photonic crystal can meet most of the characteristics of photonic crystal, and can be fabricated by planar optical waveguide technology, which has attracted extensive attention [41]. The slow-light effect of photonic crystal is very suitable for the design of phase-shifters, thus increasing the modulation efficiency. The research on silicon photonic crystal modulators started early; soon after the conventional silicon modulators demonstrated nice performance, some researchers tried to introduce the photonic crystal structure into the silicon waveguides to improve the modulation efficiency of the silicon modulators.

### 3.1. All-Silicon Photonic Crystal Modulators

**Figure 4:**
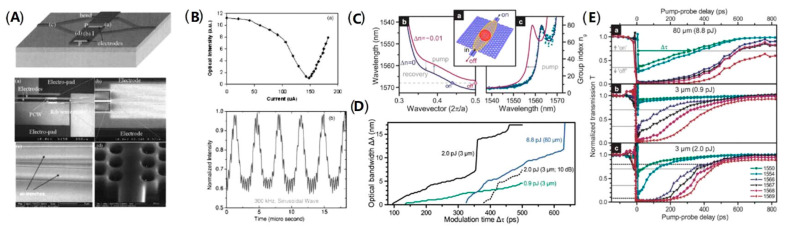
The structure and performance of silicon photonic crystal modulator. (**A**) Schematic diagram and SEM image of the silicon photonic crystal modulator [42]. (**B**) Modulation characteristics include intensity with injection current and modulation curve [42]. (**C**) Dispersion engineered photonic crystal modulator: schematic, band structure, and group index [43]. (**D**) The relationship between optical bandwidth and the modulation time [43]. (**E**) The transmission through the waveguide as a function of the time delay [43].

In 2005, Y. Jiang et al. demonstrated an ultra-compact silicon (EO) Mach–Zehnder modulator based on silicon photonic crystal waveguide for the first time, as shown in Figure 4A. The modulator has a footprint of 80 μm, and the phase shift driving current across the active region is only 0.15 mA. The 92% modulation depth is achieved at 0.15 mA, and the modulation curve for 300 kHz sinusoidal wave with a peak current of 0.11 mA is also illustrated (Figure 4B). As the first experimental demonstration, this study demonstrated the feasibility of combining photonic crystals with silicon modulator waveguides [42]. In 2013, A. Opheij et al. designed and fabricated a silicon dispersion engineered photonic crystal modulator that is only 3 μm long, based on the slow-light effect brought by photonic crystal waveguide. Figure 4C illustrates the schematic of the device with the band structure and group index of the waveguide mode. With an optical bandwidth of 7 nm, the modulation times of the modulator range between 500 ps and 100 ps. The relationship between optical bandwidth and the modulation time is shown in Figure 4D, which shows the trade-off between the two parameters. The dynamics of the modulator are demonstrated in Figure 4E, the transmission through the waveguide will change with time delay for several different wavelengths [43]. As early experimental explorations of silicon photonic crystal modulators, these researches showed the impressive advantage of silicon photonic crystal modulators especially in ultra-compact footprint and low energy consumption. However, the absolute performance of the modulators is not strong enough to satisfy the practical working requirements. 

**Figure 5:**
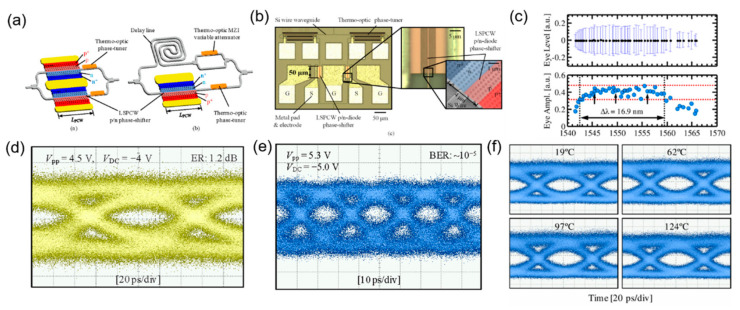
Sub−100 μm silicon photonic crystal modulator. (**a**) Schematic of the silicon photonic crystal modulators with dual and single modulation arm [44]. (**b**) Optical and SEM images of the 90 μm dual phase-shifter modulator [44]. (**c**) Spectral characteristics of the 90 μm dual silicon photonic crystal modulator [45]. (**d**) Eye diagram (10 Gb/s) of the 50 μm dual silicon photonic crystal modulator [44]. (**e**) Eye diagram (40 Gb/s) of the 90 μm single silicon photonic crystal modulator [44]. (**f**) Temperature characterization of the 90 μm dual silicon photonic crystal modulator at 10 Gb/s [45].

Taking the advantage of the compact footprint of silicon photonic crystal modulators, researchers have done a lot of work to further improve their high-speed performance under CMOS process. In 2012, H. Nguyen et al. demonstrated the silicon photonic crystal modulators with sub-100 μm length. The schemes of the two modulators with dual and single phase-shifter are shown in Figure 5a. Especially, the optical image of the 50 μm dual phase-shifter device is illustrated in Figure 5b. Based on the dual phase-shifter device with a length of only 50 μm, they obtained 10 Gb/s eye diagram of the on–off keying (OOK) over a bandwidth of 12.5 nm (Figure 5d). Meanwhile, based on the single phase-shifter device with a length of 90 μm, they achieved 40 Gb/s eye diagram (Figure 5e). The experimental results showed that silicon photonic crystal modulators can reduce the footprint by an order of magnitude compared with conventional silicon MZMs and also achieve high-speed transmission. However, the RF electrodes of the modulators were not optimized, and there is space for improvement [44]. Furthermore, they designed a 90 μm dual phase-shifter photonic crystal modulator with a spectral operating bandwidth of 16.9 nm, shown in in Figure 5c. Due to the large operating wavelength range, the modulator exhibits a good temperature stability. When the temperature changes from 19 to 124 °C, the 10 Gb/s eye diagram amplitude of the modulator is consistent within ±25%, shown in Figure 5f. This demonstrates the advantage of silicon photonic crystal modulator over silicon MRMs in terms of temperature stability, and better temperature stability of the slow-light device is the basis for the practical application [45]. 

**Figure 6:**
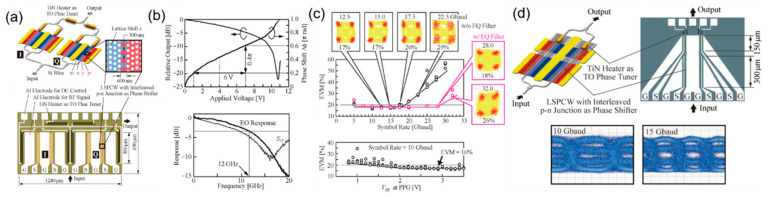
Silicon photonic crystal quadrature phase-shift keying (QPSK) and pulse amplitude modulation (PAM-4) modulator. (**a**) Scheme and optical image of the silicon photonic crystal QPSK modulator with interleaved PN junctions [46]. (**b**) Optical intensity response and frequency response of the silicon photonic crystal modulator [46]. (**c**) QPSK modulation results: EVM and constellation pattern [46]. (**d**) Silicon photonic crystal PAM-4 modulator: Scheme, optical image, and eye diagram [46].

After realizing OOK signal transmission, in order to further improve the data transmission rate, realizing the high-order signal transmission on silicon photonic crystal modulators became an important research point. Simultaneously, considering the phase modulation advantage of the Mach–Zehnder structure compared to the micro-ring structure, the coherent emission based on the slow-light modulator is also possible to realize. In 2016, K. Hojo et al. demonstrated the quadrature phase-shift keying (QPSK) constellation patterns of 56 Gb/s by a 300 μm silicon photonic crystal modulator with interleaved PN junctions, demonstrating the potential in coherent communication. The optical image of the silicon photonic crystal QPSK modulator is shown in Figure 6a, and the constellation pattern of QPSK signal is shown in Figure 6c. However, the EO bandwidth of the modulator is only 12 GHz (Figure 6b), which limits the performance of high-speed transmission. Meanwhile, due to the increased system complexity caused by the introduction of I and Q channels, the loss of the device is up to 14 dB, which still needs to be reduced in the future. Furthermore, they designed a silicon photonic crystal modulator with two segmented phase-shifters of 300 μm and 150 μm, in order to drive each section independently and obtained the four-level pulse amplitude modulation (PAM-4) signal of 30 Gb/s (15 Gbaud), shown in Figure 6d. This research proves experimentally that silicon photonic crystal modulators are capable of realizing high-order transmission and forming complex systems [46].

**Figure 7:**
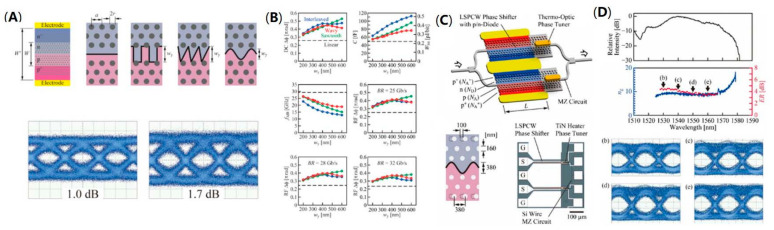
Optimized PN junctions and full C-band silicon photonic crystal modulator. (**A**) Structure of different PN junction profiles and 32 Gb/s eye diagram for wavy junction modulator [47]. (**B**) The performance comparison of four different profile junctions [47]. (**C**) The scheme and optical image of full C-band modulator [48]. (**D**) Transmission spectra, group index spectra, and 25 Gb/s eye diagrams at different wavelengths [48].

While introducing photonic crystal into the modulator waveguides to reduce the footprint, in 2017, Y. Terada et al. optimized the PN junctions by designing different periodic PN junction, as shown in Figure 7A. They theoretically analyzed the influence of the PN junctions with different profiles and parameters on device performance. Figure 7B demonstrates the performance comparison of different PN junctions including linear junction, interleaved junction, sawtooth junction, and wavy junction. Among the PN junctions with four different profiles, they found that the sawtooth and wavy junctions match with the slow-light mode to the best degree, thereby increasing the modulation efficiency. Considering the sawtooth junctions require a high-resolution process, they selected the wavy junction to fabricate the device to balance the efficiency and speed, and achieved 32 Gb/s eye diagram of OOK (Figure 7A) [47]. 

Although the introduction of the photonic crystal structure enhances the modulation efficiency, it often limits the operating wavelength range of the device, so extending the operating wavelength range of the device is an important research direction for slow-light modulators. In fact, a large operating wavelength range is critical to the practical application of the device. In 2017, Y. Terada et al. optimized the structural parameters of the photonic crystal waveguides in order to expand Δλ through decreasing the group index. As can be seen from the image of full C-band silicon photonic crystal modulator in Figure 7C, the device employs a structure of dual phase-shifters and the wavy PN junctions introduced above. After optimization, the silicon photonic crystal modulator can work with an ultra-wide Δλ of 42 nm with the group index of 8–9. With a length of 200 μm, the modulator can demonstrate 25 Gb/s eye diagram of OOK signal in the full C-band (Figure 7D). The wide operation wavelength improves the practicability of the modulator significantly. Firstly, it does not need to adjust the optimal wavelength before actual operation. Moreover, the temperature control module is not required, thereby reducing the extra energy consumption of the whole system. The wide operation wavelength is the advantage of silicon photonics modulator compared with silicon MRMs, while the length of 200 μm is also much smaller than that of conventional silicon MZMs [48]. 

**Figure 8:**
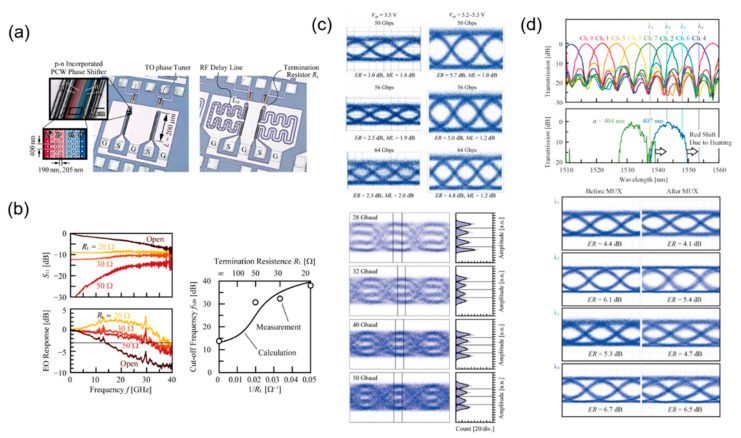
Silicon photonic crystal modulator with the meander line electrodes. (**a**) The optical image of the silicon photonic crystal modulator with the normal electrodes and the meander line electrodes [49]. (**b**) EO response of silicon photonic crystal modulator with the meander line electrodes [49]. (**c**) OOK eye diagrams of 50 Gb/s, 56 Gb/s, and 64 Gb/s and PAM4 eye diagrams of 28 Gbaud, 32 Gbaud, 40 Gbaud, and 50 Gbaud [50]. (**d**) Measurement results of WDM transmission experiment [50].

As can be seen, when the structure of photonic crystal is introduced to the modulator, the slow-light effect will increase the modulation efficiency, and hold a wide working spectrum in the meantime. However, the phase mismatch between slow light and RF signals limits the high-frequency performance of the modulator. To overcome this issue, in 2019, Y. Hinakura et al. designed the meander line electrodes that can delay RF signals to reduce the electrooptic phase mismatch between slow light and RF signals. The structure comparison between the normal electrodes and the meander line electrodes is demonstrated in Figure 8a. Through this approach, the EO bandwidth reaches 31 GHz and 38 GHz experimentally according to the termination resistors adopted (Figure 8b) [49]. Furthermore, using the meander line electrodes, they demonstrated a silicon photonic crystal modulator, which has the modulation efficiency of 0.44 V·cm, a working spectrum over 15 nm, and a length of 200 μm. Especially, based on the excellent high-frequency performance and modulation efficiency of the device, the improvement in signal transmission is impressive. A 64 Gb/s OOK signal and a 100 Gb/s PAM-4 are realized (Figure 8c), while a 4 × 50 Gb/s wavelength division multiplexing using an additional MUX chip is demonstrated (Figure 8d) [50]. Overall, the silicon photonic crystal modulator has the advantages of compact footprint, low cost, high speed, and wide working spectrum, demonstrating an important application prospect in the next-generation network.

### 3.2. Silicon-Based Hybrid Photonic Crystal Modulators

While the all-silicon photonic crystal modulators demonstrate impressive performance due to the slow-light effect, especially in the aspect of high modulation efficiency and compact footprint, the silicon-based photonic crystal structure can also be integrated with other materials to realize high-performance EO modulation, such as LN, polymer, and graphene. The silicon-based hybrid photonic crystal modulators combine the excellent EO properties of other materials and the slow-light effect introduced by photonic crystal together, and are expected to achieve higher breakthroughs in performance.

**Figure 9:**
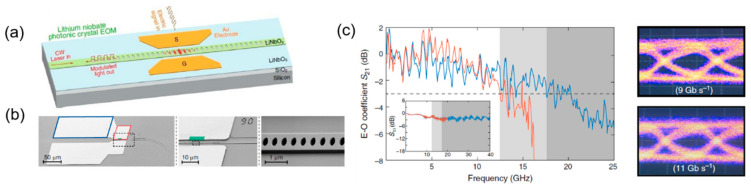
Silicon-based lithium niobate (LN) photonic crystal modulator (**a**) Schematic of the designed modulator [52]. (**b**) SEM image of the detailed structure [52]. (**c**) The high-speed performance of the modulator: electro-optic bandwidth and eye diagram [52].

Recently, the thin-film LN-on-insulator platform has emerged as a promising candidate for integrated high-performance EO modulators [51]. Based on this platform, ultrahigh EO bandwidth and low loss have been achieved [16]. However, they usually suffer from a large footprint, which impedes the dense integration. To solve this limitation, in 2020, Li. M et al. applied a photonic crystal waveguide structure for an LN modulator on silicon substrate. The design schematic is shown in Figure 9a, and the SEM image of the fabricated device is shown in Figure 9b. Through controlling the light confinement and light–matter interactions on a subwavelength scale, the device size has been greatly reduced into a small footprint of only 0.58 μm^3^. Meanwhile, this photonic crystal waveguide LN modulator demonstrates an EO bandwidth of 12.5 GHz and achieves an eye diagram of 11 Gb/s (Figure 9c) with a bit-switching energy as low as 22 fJ [52].

**Figure 10:**
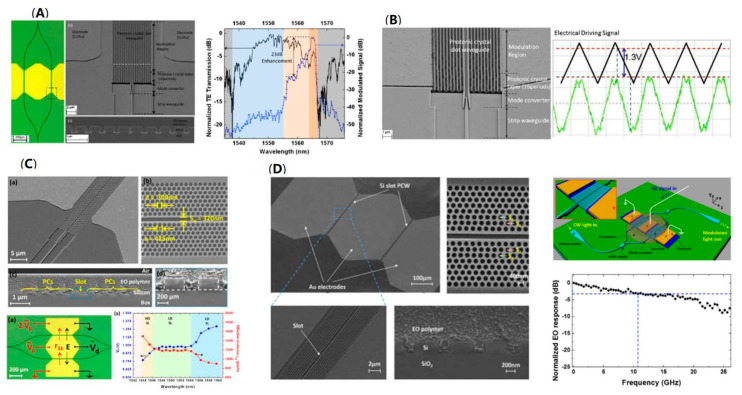
Silicon-based polymer photonic crystal modulator. (**A**) Optical and SEM images of the polymer-infiltrated silicon photonic crystal slot modulator and its wavelength dependence of normalized modulated signal (blue line) and normalized optical transmission (black line) [55]. (**B**) SEM picture of the 320 nm-wide silicon photonic crystal slot waveguide. The modulation measurements showing a low Vπ of 1.3 V [56]. (**C**) SEM images of the band-engineered, EO polymer-refilled silicon slot photonics crystal modulator. Measured Vπ and corresponding calculated effective r_33_ versus wavelength (at 100 kHz) [57]. (**D**) SEM images of the polymer-refilled photonic crystal modulator with RC time constant engineered and a backside gate technique. Measured normalized EO response of the modulator as a function of RF frequency indicates a 3 dB modulation bandwidth of 11 GHz [58].

The polymer, characterized with the advantages of large EO coefficient, ultrafast response time, low dispersion, and spin-coating feature, is regarded as an ideal material for the EO modulators with low power consumption, ultra-high-speed operation, and ease of fabrication [53]. Hybrid integration of the silicon-based photonic crystal waveguides with electro-optic polymer realizes high-performance ultra-compact optical modulators through the combination of the slow-light effect and the unprecedented EO properties of polymers. In 2008, the electro-optic modulator with a polymer-infiltrated silicon photonic crystal waveguide was firstly proposed, which achieves a bandwidth of 78 GHz, a drive voltage amplitude of 1 V, and a length of only 80 μm [54]. Several physical effects were exploited for the remarkable performance, including the fast and strong nonlinearities of polymers infiltrated into silicon, the long interaction time provided by the Mach–Zehnder interferometer with slotted slow-light waveguides, and the short modulator length boosting the RC-constant-limited bandwidth [54]. Subsequently, such an EO modulator was demonstrated experimentally. By hybrid integration of the EO polymer with photonic crystal waveguide, an ultra-efficient EO modulation with a record low modulation efficiency of 0.56 V·mm was demonstrated. The modulated signal shows strong wavelength dependence and peak enhancement of 23 dB (Figure 10A) [55]. Nevertheless, the in-slot EO efficiency is relatively low due to the degrading poling efficiency, hindered by the narrow slot. A 320 nm slot for an electro-optic polymer-infiltrated silicon photonic crystal waveguide is demonstrated to overcome the problem. An effective in-device r_33_ of 735 pm/V as well as VπL = 0.44 V·mm were achieved (Figure 10B) [56]. A narrow operating optical bandwidth of <1 nm is an important problem remaining among slot photonic crystal modulators because of the high group-velocity dispersion in the slow-light optical spectrum range. To broaden the operating optical bandwidth of photonic crystal modulators, X. Zhang et al. demonstrated the lattice-shifted photonic crystal waveguides. The spatial shift of certain holes provides low-dispersion slow light and realizes an optical operation range of 8 nm (Figure 10C) [57]. A modulation efficiency of Vπ L = 0.282 V·mm measured at 100 KHz is achieved by slow-light enhancement, which realizes a record high effective in-device EO coefficient (r_33_) of 1230 pm/V. In addition, by introducing the silicon doping and backside gate technique, the EO bandwidth of the device is measured to be 15 GHz (Figure 10D) [58].

**Figure 11:**
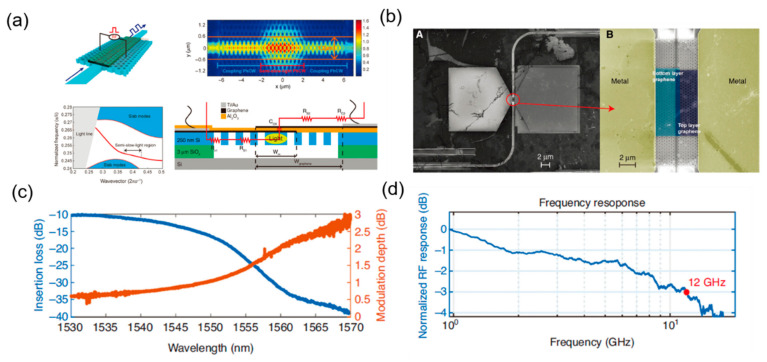
Silicon-based graphene photonic crystal modulator. (**a**) Structure, photonic band diagram, electric field distribution, and equivalent circuit of the graphene modulator [62]. (**b**) SEM image of the fabricated double-layer graphene modulator [62]. (**c**) Insertion loss and modulation depth with the change of wavelength [62]. (**d**) Frequency response of the silicon-based graphene photonic crystal modulator [62].

Integrating 2D materials such as graphene to silicon devices has proved to be an effective way to improve the performance of the silicon-based EO modulator, including modulation speed and efficiency [18,59]. The EO modulation realized by electron accumulation makes the graphene EO modulator a promising candidate for low-temperature-environment application and long-haul communication [60,61]. The graphene is also integrated with photonic crystal waveguide to increase the light–matter interaction. In a recent work, a double-layer graphene electro-absorption modulator was demonstrated in telecommunication applications, as shown in Figure 11a,b, which achieves a modulation of 1.5 dB at 1555 nm (Figure 11c) and a modulation bandwidth at 12 GHz (Figure 11d) [62].

## 4. Silicon Waveguide Grating Modulators

However, for two-dimensional photonic crystal, the fabrication is still relatively challenging under the standard CMOS manufacturing process, which has relatively higher requirements on the process. The photonic crystal structure has a low tolerance to the fabrication process. If there is a small deviation in the parameters during the process, the performance will change obviously, and the structural quality is indeed prone to deviation in the fabrication, thus causing difficulty for large-scale wafer manufacturing [63]. In order to avoid process limitations and take the advantage of CMOS compatibility for silico modulators, silicon slow-light modulators based on waveguide gratings have been proposed and fabricated theoretically and experimentally in recent years.

### 4.1. All-Silicon Waveguide Grating Modulators

**Figure 12:**
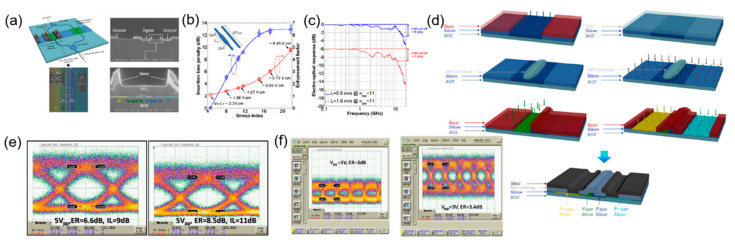
The structure and performance of silicon waveguide grating modulator. (**a**) Schematic and SEM of silicon waveguide grating modulator [64]. (**b**) The relationship between modulation efficiency and group index of the slow-light modulator [64]. (**c**) EO response of two modulators with slow-light phase-shifter lengths of 0.5 mm and 1 mm [64]. (**d**) CMOS fabrication process for the silicon waveguide grating modulator [66]. (**e**) Eye diagram of 40 Gb/s for 500 μm device [65]. (**f**) Eye diagram of 5 Gb/s and 25 Gb/s for 1 mm device at low drive voltage [66].

In 2011, A. Brimont et al. demonstrated a silicon slow-light modulator with Mach–Zehnder interferometer to explore the effects of introducing grating structures into silicon modulator waveguides (Figure 12a). The slow-light effect changes the group index, and they illustrated that the modulation efficiency changes with the group index experimentally. In fact, the modulation efficiency of the modulator is enhanced by the increase of the group index. When the group index increases up to 22, the modulation efficiency achieves a value of 0.45 V·cm, as shown in Figure 12b. Meanwhile, the EO bandwidth of two modulators with slow-light phase-shifter lengths of 0.5 mm and 1 mm can reach 16 GHz and 11 GHz, respectively (Figure 12c) [64]. For slow-light phase-shifters of 500 μm, the modulator achieves an eye diagram of 40 Gb/s with 6.6 dB extinction ratio at quadrature (Figure 12e), with an enhanced modulation efficiency of 0.85 V·cm and on-chip insertion loss of 6 dB [65]. For 1 mm slow-light phase-shifters, the high modulation efficiency of 0.6 V·cm allows the modulator to work from 5 Gb/s with 1 Vpp up to 25 Gb/s with 3 Vpp, shown in Figure 12f. The low drive voltages make the device more suitable for the application of CMOS transceivers, but the insertion loss is relatively high, reaching 12 dB [66]. It can be seen that the modulation efficiency has been significantly improved, thus the footprint can be reduced to the order of several hundred microns, and achieve a favorable eye diagram at high speed, while containing the length of 1 mm is beneficial for achieving a low drive voltage. It is worth noting that the waveguide grating modulator is very suitable for manufacturing under CMOS fabrication process, and the specific procedures are illustrated in Figure 12d [66]. As early explorations into the introduction of waveguide grating structure in silicon modulators, these results demonstrate experimentally that the slow-light effect brought by waveguide grating can enhance the performance of silicon modulators under CMOS fabrication process.

**Figure 13:**
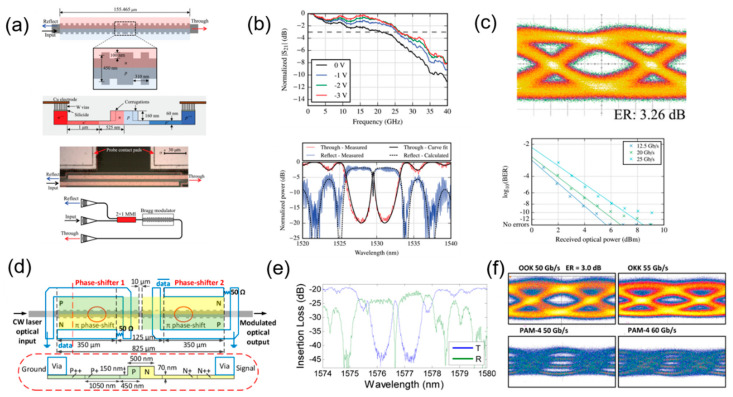
The structure and performance of silicon modulator with Bragg grating. (**a**) Schematic and optical image of the silicon modulator with quarter-wave phase-shifted Bragg grating resonator [67]. (**b**) EO bandwidth and spectra of the quarter-wave phase-shifted Bragg grating modulator [67]. (**c**) OOK of 32 Gb/s and BER curve of the quarter-wave phase-shifted Bragg grating modulator [67]. (**d**) Schematic of the dual phase-shift Bragg grating modulator [68]. (**e**) Spectra at different voltages of the dual phase-shift Bragg grating modulator [68]. (**f**) OOK and PAM-4 eye diagram of the dual phase-shift Bragg grating modulator [68].

In 2015, M. Caverley et al. reported a silicon modulator with quarter-wave phase-shifted Bragg grating resonator on the SOI platform. With a length of 155 μm, the modulator has two ports, through port and reflect port, shown in Figure 13a. The modulated output signal is mainly obtained from the reflect port, which has a spectral response of a sharp notch, and the modulator demonstrates the EO bandwidth of 26 GHz (Figure 13b), and achieves the OOK eye diagram of 32 Gb/s with a bit error ratio (BER) of less than 10^−10^ of 25 Gb/s, as shown in Figure 13c. However, the spectrum of the modulator is very narrow, and its operation is affected by the wavelength, which affects its practical application [67]. In 2016, K. Bédard et al. demonstrated a dual phase-shift silicon modulator with Bragg grating structure, as shown in Figure 13d. The silicon waveguide grating modulator achieves an OOK signal up to 55 Gb/s with MMSE equalization and up to 50 Gb/s without equalization, while PAM-4 signal is illustrated at 60 Gb/s (Figure 13f). However, although the modulation speed is high, this modulator has a long length of 825 μm with a narrow spectrum (Figure 13e), which is insufficient to illustrate advantages compared to conventional silicon MZMs and MRMs [68]. 

It can be seen that the slow-light effect introduced by the grating structure in the silicon modulators improves the modulation efficiency, thereby reducing the energy consumption. In order to study the effect of silicon modulator assisted by waveguide grating on reducing energy consumption specifically, in 2018, R. Hosseini et al. investigated the slow-light effect on improving the energy efficiency of the modulator and compared it with conventional methods by increasing doping concentration. Considering the trade-off between the modulation efficiency and loss, the loss–modulation efficiency product figure of merit was proposed. Under the condition of the similar energy reduction, the loss-modulation efficiency product of the slow-light waveguide is smaller than that of the high-doping modulator. The research has shown the ability of the slow-light structure to reduce the energy consumption theoretically [69]. However, the analysis of the model is not comprehensive enough, and the influence of the waveguide parameters on various performance parameters of the modulator is not illustrated in detail. 

**Figure 14:**
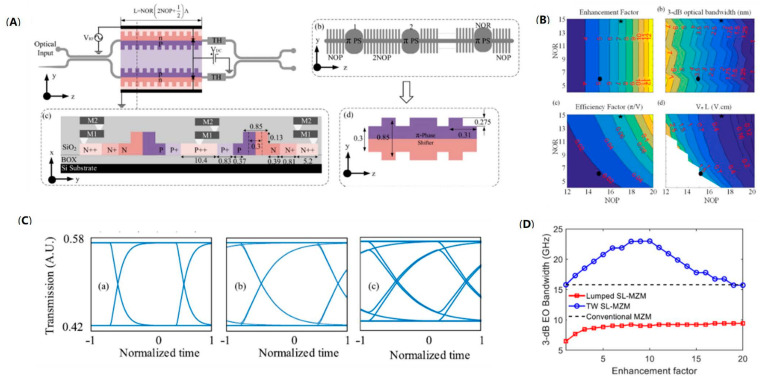
The model of silicon Bragg grating modulator. (**A**) Schematic of the silicon Bragg gratings modulator [70]. (**B**) Enhancement factor γ, optical bandwidth, efficiency factor, and modulation efficiency with the change of NOP and NOR [70]. (**C**) Eye diagram simulated at 30 Gb/s, 70 Gb/s, and 110 Gb/s [70]. (**D**) EO bandwidth as a function of enhancement factor for silicon Bragg gratings modulator with different electrodes [71].

Due to the introduction of the grating structure into the modulator waveguide, the parameters and period of the grating will have a significant impact on the performance of the modulator. Through establishing a quantitative model, the different parameters of the grating will be related to the performance of the modulator directly. Therefore, establishing a precise simulation model of a silicon slow-light modulator assisted with waveguide grating will guide the specific design. In 2019, O. Jafari et al. demonstrated the design of silicon integrated Bragg grating resonators modulator. In each arm of the Mach–Zehnder interferometer, a series of Bragg gratings resonators are introduced to enhance the phase modulation ability based on slow-light effect, as shown in Figure 14A. Therefore, the designed silicon Bragg gratings modulator has stronger phase modulation capability than that of conventional silicon MZMs, while holding a large optical bandwidth compared to silicon MRMs. They established a complete theoretical model to explore the effect of different Bragg parameters, including NOP and NOR (NOP: number of periods of the resonator mirrors on each side; NOR: number of resonators), on the device performance. Therefore, the modulator was optimized according to the modulation efficiency, efficiency factor, enhancement factor, and optical bandwidth (Figure 14B). Through the simulation modeling, the variation law of the performance of the device with the structural parameters was more completely and intuitively reflected, which has reference significance for the practical design of the device. In particular, according to the dynamic response model of the modulator based on the coupled-mode theory, the large-signal analysis was performed using the finite-difference time domain and the OOK eye diagram up to 110 Gb/s, simulated in Figure 14C, showing the ultra-high-speed operation potential of the modulator [70]. Furthermore, focus on the efficiency–speed tradeoff in silicon slow-light modulators, they established a comprehensive model for the EO response of lumped-electrode slow-light modulators. Meanwhile, for the silicon slow-light modulators with traveling-wave electrodes, they used the finite-difference time-domain method to show that the efficiency and speed can both be improved under an optimized slow-light effect. The relationship between the EO bandwidth and enhancement factor is demonstrated in Figure 14D. However, the additional loss caused by slow-light waveguide limits the application of such modulators [71]. 

**Figure 15:**
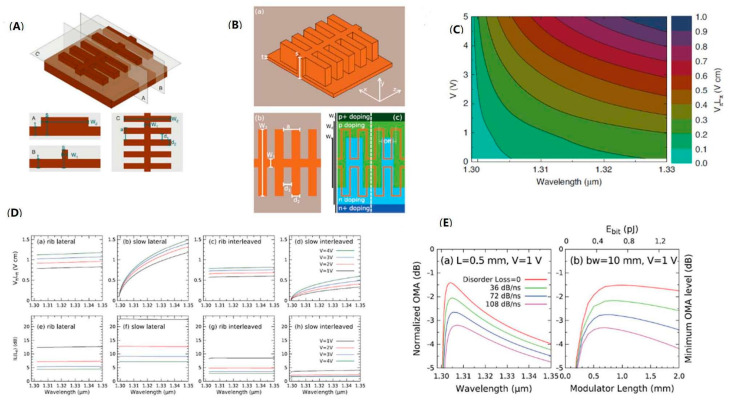
The model of silicon waveguide grating modulator with interleaved PN junction. (**A**) Structure of the slow-light waveguide grating [72]. (**B**) Schematic of the slow-light waveguide with interleaved PN junctions [73]. (**C**) Modulation efficiency change with wavelength and bias voltage [72]. (**D**) The comparation of VπLπ, IL (Lπ) for phase-shifters in four conditions: rib waveguide with lateral PN junction; slow-light waveguide with lateral PN junction; rib waveguide with interleaved PN junction; slow-light waveguide with interleaved PN junction [73]. (**E**) Normalized OMA variation with different parameters of the silicon slow-light modulator [73].

Meanwhile, optimizing the PN junctions is also the method to improve the modulation efficiency for the silicon modulator. The modulation efficiency can be improved by introducing interleaved PN junction, and combining these two structures to further optimize silicon modulators has also become an attractive research point. In 2019, M. Passoni analyzed this composite structure theoretically. Based on the silicon slow-light modulator with the waveguide grating structure in Figure 15A, the interleaved PN junctions with the same period as the grating were introduced into the modulation arm, to achieve optimal matching between the electromagnetic field and the depletion regions of the PN junction, thereby further improving the performance of the modulator. The bandwidth of the modulator was increased by the interleaved PN junction, because the spatial matching between the field of the grating and the depletion region is independent for wavelength. According to the simulation results, the modulation efficiency was improved compared to the conventional silicon modulator, with 0.1–0.5 V·cm over a bandwidth of 20–30 nm, as shown in Figure 15C. The research illustrates the trade-off between modulation rate, loss, and energy consumption. However, the optimization of the device was not enough, and further optimization should be able to get better results [72]. Furthermore, they developed the full simulation of the silicon slow-light modulator with interleaved PN junction along the waveguide axis, as in Figure 15B. The simulation investigated the optical modulation amplitude (OMA), which accounts for loss and modulation efficiency, further elaborating the trade-off among modulation efficiency, cutoff frequency, optical modulation amplitude, insertion loss, and dissipated energy per bit. Figure 15D demonstrates the comparison of modulation efficiency, IL (Lπ), for phase-shifters in four different conditions. After introducing the structure of the interleaved PN junction, the optical bandwidth is wider than the slow-light bandwidth. Under the condition of 1 V reverse bias, the modulator can achieve an energy below 0.5 pJ/bit and a bandwidth of tens of nanometers with a length below 0.5 mm (Figure 15E). This simulation demonstrates that a suitable design of interleaved PN junction will enhance the performance of silicon slow-light modulator for reducing power dissipation [73]. 

**Figure 16:**
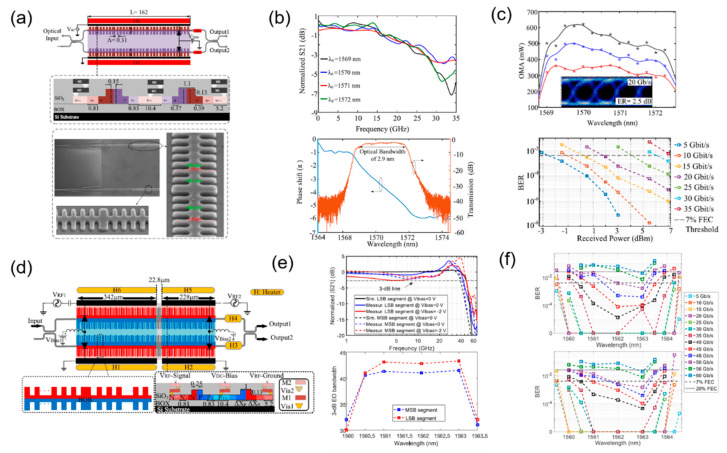
The structure and performance of silicon slow-light modulator with integrated Bragg grating resonators. (**a**) Schematic, cross-section and SEM picture of the modulator [74]. (**b**) The transmission amplitude and EO response of the slow-light modulator [74]. (**c**) OMA and BER measured for 10 Gb/s, 20 Gb/s, and 30 Gb/s with eye diagram of 20 Gb/s [74]. (**d**) Schematic of silicon segmented slow-light modulator [75]. (**e**) EO responses and 3 dB EO bandwidth for two segments of the modulator [75]. (**f**) BER spectral for PAM-4 of the DAC-less modulator [75].

On the basis of the previous exploration of simulation research, the practical experimental performance research of silicon waveguide grating modulators has also made progress in recent years. In 2020, O. Jafari et al. demonstrated a silicon slow-light modulator assisted by phase-shifted Bragg gratings experimentally, which was fabricated under CMOS process, shown in Figure 16a. The integrated Bragg grating resonators enhanced the phase modulation efficiency significantly, and a small-signal Vπ × L of 0.18 V·cm was obtained, which is an extremely high value for an all-silicon modulator. These experimental data prove the significant effect of the slow-light structure on improving the modulation efficiency of the modulator. Meanwhile, the modulator shows an EO bandwidth of 28 GHz and an optical bandwidth of 2.9 nm (Figure 16b), with a length of 162 μm, which is much smaller than that of conventional silicon MZMs. The optical bandwidth is larger than that of the silicon modulators with waveguide grating reported before, which should provide an operating temperature range larger than 40 °C and illustrate the advantage compared to silicon MRMs. Meanwhile, OOK modulation is demonstrated at 30 Gb/s with a BER below the 7%-overhead FEC threshold in Figure 16c. However, in general, although the modulation efficiency and footprint of the modulator have both achieved ideal values, the EO bandwidth of the modulator is still not large enough and the high-speed performance is limited, while the optical bandwidth also has a certain space for optimization for a more stable working condition [74]. 

Furthermore, based on the slow effect introduced by the structure of integrated Bragg grating resonators above, a segmented slow-light modulator was designed for PAM-4 signal transmission (Figure 16d). This segmented modulator has a compact footprint of 570 μm, low energy consumption of 73 fJ/bit, high modulation efficiency of 0.51 V·cm, large EO bandwidth of 40 GHz up, as shown in Figure 16e. A high-speed PAM-4 signal is generated by driving the segmented phase-shifter without an electrical digital-to-analogue converter (DAC), and 90 Gb/s PAM-4 is achieved over a spectral operation of 2 nm, shown in Figure 16f. This experimental result demonstrates the potential of silicon waveguide grating modulators to transmit complex signal, and also illustrates the possibility of co-designing silicon waveguide grating modulators as a unit for complex functions under CMOS fabrication process [75]. 

**Figure 17:**
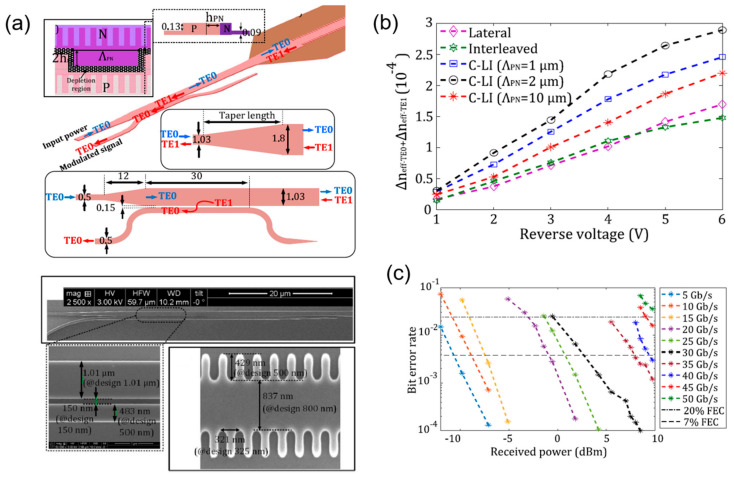
The structure and performance of silicon mode-conversion modulator with asymmetric Bragg grating. (**a**) Schematic and SEM of the modulator structure [76]. (**b**) Phase-shift of the asymmetric Bragg grating modulators with PN junctions of different profiles [76]. (**c**) BER for different modulation speeds [76].

Meanwhile, in 2021, O. Jafari et al. designed a silicon mode-conversion modulator, combing the structure of asymmetric Bragg grating with lateral and interleaved PN junctions (C-LI) to enhance the phase modulation ability, as shown in Figure 17a. Under the mode conversion brought by an asymmetric Bragg grating, the modulator can work in reflection mode. Meanwhile, after employing the lateral and interleaved PN junction, the modulator gains a 67% improvement in the phase modulation, as shown in Figure 17b. The modulator achieved 45 Gb/s with a BER below the 7% forward-error-correction (FEC) threshold and 55 Gb/s with 20% (Figure 17c). The modulator has a length of 290 μm, a low loss of 2 dB, and a low power consumption of 226 fJ/bit, but the EO bandwidth is only 11.2 GHz, which limited the high-speed performance. Although the absolute performance of this modulator is limited, the idea of introducing mode conversion based on Bragg gratings and different PN junction profiles is instructive, which will also generate inspiration for designing other new devices based on the effects introduced by grating structures [76]. 

### 4.2. Silicon-Based Hybrid Waveguide Grating Modulators

As discussed above, all-silicon waveguide grating modulators demonstrated the advantages of compact footprint, high modulation efficiency, and low energy consumption. Meanwhile, the silicon-based hybrid waveguide grating modulator has been investigated to enhance the performance further based on the properties of other material and the benefits of slow-light effect, such as hybrid lithium niobate waveguide grating modulator. 

**Figure 18:**
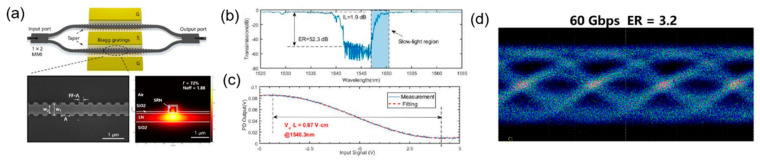
Silicon-based lithium niobate waveguide grating modulator. (**a**) Schematic and SEM of the thin-film lithium niobate modulator [77]. (**b**) Transmission spectra of the device with Bragg grating waveguides [77]. (**c**) Modulation response of the slow-light modulator [77]. (**d**) Eye diagram (60 Gb/s) of the slow-light modulator [77].

In 2021, X. Huang et al. reported a sub-millimeter-long hybrid silicon-rich nitride and thin-film lithium niobate modulator based on Bragg grating waveguides. This modulator is based two 800 μm Bragg grating waveguides, which serve as phase-shifters (Figure 18a). By operating at the band edge of Bragg grating, the modulation efficiency is greatly enhanced with slow-light effect, increasing the light–matter interaction time. Using this modulator, an insertion loss of 1.9 dB (Figure 18b) and a modulation efficiency of 0.67 V·cm (Figure 18c) were experimentally demonstrated with 60 Gb/s data transmission (Figure 18d) [77]. This study demonstrates that the silicon-based lithium niobate waveguide grating modulator has the potential to achieve high modulation efficiency and compact footprint while maintaining the high-speed performance of thin-film lithium niobate modulators.

However, up to now, research relating to the silicon-based hybrid waveguide grating modulators with other materials such as polymer and graphene is limited. Fabricating waveguide gratings of these materials to construct modulators on silicon substrate requires more efforts of the researchers.

## 5. Discussion

Table 1 lists the experimental results of representative silicon slow-light modulators under CMOS process and compares their performance. Since CMOS compatibility is the core advantage of silicon slow-light modulators, we compare the experimental performance of all-silicon slow-light modulators in the table to demonstrate the potential of all-silicon modulation under CMOS process. As can be seen, silicon photonic crystal modulators are superior in the aspect of transmission rates and transmission format complexity, especially the operating wavelength range is far beyond that of silicon waveguide grating modulators, while silicon waveguide grating modulators generally have lower loss than that of silicon photonic crystal modulators, and are relatively easier to manufacture in a large-scale wafer-level CMOS process. As for the EO bandwidth, they both demonstrate certain limitations, and there exits potential for improvement. In terms of modulation efficiency, both of them are significantly improved due to the slow-light effect, and the device footprint is relatively close and significantly smaller than that of the conventional silicon MZMs. While holding a nice high-speed transmission performance, the modulation efficiency is improved and the footprint is reduced significantly due to slow-light effect, which fully demonstrates the potential in high-density optoelectronic integration.

Under the premise of ensuring high-speed performance and large optical bandwidth, the silicon slow modulators achieve a further reduction in footprint. In fact, the compact footprint of the silicon slow-light modulators is suitable especially for applications such as artificial intelligence, on-chip transmission, and large-scale optoelectronic integration, which require large-scale modulator arrays. Taking a practical application scenario as a specific application suggestion, we believe that the integration with CMOS drivers would be ideal for silicon slow-light modulators. In fact, the integration of conventional silicon MZMs with CMOS drivers is always constrained by footprint mismatch between silicon MZMs and CMOS drivers. After the utilization of silicon slow-light modulators, the device footprint is able to reduce to the level of microelectronics devices, which will benefit the connection and package between the modulators and drivers particularly. In the meanwhile, due to the large optical bandwidth, the silicon modulators exhibit a nice thermal stability compared to silicon MRMs, while also possessing a compact footprint. This means that the silicon modulators can hold a stable operation status in high-density transceivers without the utilization of a temperature control module.

In order to further improve the performance of silicon slow-light modulators and satisfy the application requirements in practical silicon optoelectronic integrated systems, efforts are needed in the following major directions in the future:

1. Complex format transmission: The primary advantage of silicon slow-light modulators is their ultra-compact footprint, while silicon MRMs are also with an advantage in size, so compared with the silicon MRMs, the advantages of function for the slow-light modulator need to be demonstrated clearly. The silicon slow-light modulator is conducive to realizing phase modulation, thereby transmitting signals such as BPSK and QPSK, and realizing the fabrication of coherent transmitters. Although there exists research on silicon photonic crystal modulators to obtain QPSK constellation pattern, the research on high-order data transmission is relatively limited, and there is still no phase modulation experiment for silicon waveguide grating modulators. Therefore, developing the complex signal transmission function of silicon slow-light modulators to realize silicon coherent transmitters of slow light is an important research direction.
2. System integration: The ultra-compact footprint of silicon slow-light modulators has intrinsic advantages in further optoelectronic integration, but currently there are few silicon system integrations based on slow-light modulators. Large-scale system integration is an important direction for the further development of phonics chip and optical communication systems. Practically, in the current silicon optoelectronics integrated systems, conventional silicon MZMs are always the main limiting factor. If the silicon slow-light modulator an order of magnitude smaller than the conventional silicon MZMs can be used as a unit device, the area of the system integration chip will be reduced significantly and the advantages of slow-light modulators will be maximized.
3. Reduce loss: Although the loss of the silicon slow-light modulators is not particularly high compared to that of the conventional silicon modulators, it is based on an extremely short length. Indeed, the silicon slow-light modulator is sensitive to the length of slow-light waveguide. If the waveguide length increases, the loss will increase accordingly. This condition limits the length of the silicon slow-light modulators. However, if the length of the silicon slow-light modulators can be increased enough on the premise of controlling the loss, according to the extremely high modulation efficiency brought by slow-light effect, a very low half-wave voltage will be obtained, which is very suitable for optoelectronic integration.

Silicon-based slow-light electro-optic modulators exhibit a series of advantages, such as compact footprint, low power consumption, large optical bandwidth, and CMOS compatibility, which is an important technical solution to break through the key issue of energy consumption and volume required by complex systems in future optoelectronic integration. With the continuous efforts of researchers, the silicon-based slow-light electro-optic modulators will achieve further progress as the core unit device, leading to significant development in the field of silicon photonics.

## Figures and Tables

**Table 1:** Comparison for experimental results of representative silicon slow-light modulators.

Ref.	Structure	Footprint	EO Bandwidth	Optical Bandwidth	Modulation Efficiency	Loss	Speed
[44]	Photonic crystal	50 μm/ 90 μm	NA	12.5 nm/2 nm	NA	9.1 dB/6.2 dB	10 Gb/s OOK 40 Gb/s OOK
[45]	Photonic crystal	90 μm	NA	16.9 nm	NA	8 dB	40 Gb/s OOK
[46]	Photonic crystal	300 μm/ 450 μm	12 GHz	NA	0.32 V·cm	14 dB	56 Gb/s QPSK 30 Gb/s PAM-4
[48]	Photonic crystal	200 μm	NA	42 nm	NA	4–5 dB	25 Gb/s OOK
[49]	Photonic crystal	200 μm	31 GHz/ 38 GHz	15 nm	0.6 V·cm	6–8 dB	64 Gb/s OOK
[50]	Photonic crystal	200 μm	32–38 GHz	15 nm	0.44 V·cm	6 dB	64 Gb/s OOK 100 Gb/s PAM-4 4 × 50 Gb/s WDM
[64]	Waveguide grating	500 μm/ 1000 μm	16 GHz/ 11 GHz	NA	0.45 V·cm	13 dB (1000 μm)	40 Gb/s OOK 30 Gb/s OOK
[65]	Waveguide grating	500 μm	NA	1.3 nm	0.85 V·cm	6 dB	40 Gb/s OOK
[66]	Waveguide grating	1000 μm	NA	NA	0.6 V·cm	12 dB	25 Gb/s OOK
[67]	Waveguide grating	155 μm	26.5 GHz	NA	NA	45.3 dB/cm	32 Gb/s OOK
[68]	Waveguide grating	825 μm	NA	NA	NA	2.8 dB	55 Gb/s OOK 60 Gb/s PAM-4
[74]	Waveguide grating	162 μm	28 GHz	2.9 nm	0.18 V·cm	2 dB	30 Gb/s OOK
[75]	Waveguide grating	570 μm	>40 GHz	2 nm	0.51 V·cm	5.5 dB	90 Gb/s PAM-4
[76]	Waveguide grating	290 μm	11.2 GHz	NA	NA	2 dB	55 Gb/s OOK
